# Head versus heart: social media reveals differential language of loneliness from depression

**DOI:** 10.1038/s44184-022-00014-7

**Published:** 2022-10-18

**Authors:** Tingting Liu, Lyle H. Ungar, Brenda Curtis, Garrick Sherman, Kenna Yadeta, Louis Tay, Johannes C. Eichstaedt, Sharath Chandra Guntuku

**Affiliations:** 1 National Institute on Drug Abuse (NIDA IRP), National Institutes of Health (NIH), Baltimore, MD USA; 2 Positive Psychology Center, University of Pennsylvania, Philadelphia, PA USA; 3 Department of Computer and Information Science, University of Pennsylvania, Philadelphia, PA USA; 4 Department of Psychological Sciences, Purdue University, West Lafayette, IN USA; 5 Department of Psychology, Institute for Human-Centered A.I., Stanford University, Stanford, CA USA

**Keywords:** Psychology, Depression, Risk factors, Computer science

## Abstract

We study the language differentially associated with loneliness and depression using 3.4-million Facebook posts from 2986 individuals, and uncover the statistical associations of survey-based depression and loneliness with both dictionary-based (Linguistic Inquiry Word Count 2015) and open-vocabulary linguistic features (words, phrases, and topics). Loneliness and depression were found to have highly overlapping language profiles, including sickness, pain, and negative emotions as (cross-sectional) risk factors, and social relationships and activities as protective factors. Compared to depression, the language associated with loneliness reflects a stronger cognitive focus, including more references to cognitive processes (i.e., differentiation and tentative language, thoughts, and the observation of irregularities), and cognitive activities like reading and writing. As might be expected, less lonely users were more likely to reference social relationships (e.g., friends and family, romantic relationships), and use first-person plural pronouns. Our findings suggest that the mechanisms of loneliness include self-oriented cognitive activities (i.e., reading) and an overattention to the interpretation of information in the environment. These data-driven ecological findings suggest interventions for loneliness that target maladaptive social cognitions (e.g., through reframing the perception of social environments), strengthen social relationships, and treat other affective distress (i.e., depression).

## Introduction

Loneliness, defined as the emotional unpleasant experience when individuals perceive their interpersonal relationships are not up to their expectations^1–3^, is highly prevalent in the general population and people with mental health disorders^3–5^. Loneliness impacts psychological functioning and predicts increased morbidity and mortality^2,6,7^. Multiple prior studies in the psychology, public health, and psychiatry domains report that loneliness is highly correlated with depression (e.g., Pearson’s *r* of 0.4–0.6^5,8–13^), a common mental disorder characterized by persistent sadness, disturbed sleep and appetite, and anhedonia^14^. Therefore, loneliness is often investigated as a transdiagnostic facilitator together with depression or considered as an adjunct to the treatment of depression^5,8–13^ and has been overlooked during clinical encounters^6,15^. To develop interventions specifically targeted at reducing loneliness, we need to distinguish the unique markers of loneliness from depression. The current study uses both psychological assessments and language from Facebook posts to shed light on the distinctive markers of loneliness.

The relationship between loneliness and depression appears to be reciprocal in nature. In studies on causes, consequences, and treatment plans for loneliness, depression is frequently considered to be an important influential factor and a wide-known pathological consequence^16,17^. In studies on depression, loneliness is identified as a risk factor and subsymptom for depression^9,18^. Both loneliness and depression have been linked to social skills deficits, maladaptive social cognition, internalizing distress, and uncontrollable thoughts, including worries and ruminating thoughts^19–21^. In contrast to the well-discussed association between loneliness and depression, limited attention has been given to understanding the unique markers of loneliness from depression. Initial evidence indicates that loneliness and depression are two related yet separate constructs^22^. For example, a 5-year longitudinal survey study showed that loneliness and depression are statistically separable, and loneliness predicted subsequent changes in depressive symptoms^17^. In addition, other studies suggest that loneliness has its unique symptomology^23^. Even for the mechanisms shared by loneliness and depression, recent findings from self-report surveys suggest that variance emphasis on uncontrolled thoughts and worries might distinguish loneliness from depression^19^. Specifically, loneliness has a unique function in cognition by providing implicit hypervigilance for social threats^24^, although such cognitive bias in the social context was initially evolved to adversely protect individuals from social threats and meaningless social interactions^25^.

It is important to understand such differential features associated with loneliness^26^. Considering the strong association between loneliness and depression, loneliness is often overshadowed in research on depression (e.g., as a subsymptom^18,27^). Such vagueness might lead to the unsuccessful choice of intervention types that target different components in reducing loneliness (e.g., improving social skills, increasing social supports, improving social cognition abilities, etc.)^20^, and might also result in reducing the efficacy of interventions on loneliness. More importantly, loneliness is uniquely linked to negative feelings associated with perceptions of expectations about social relationships^25^, an inherently social need of being human. Being lonely may lead to perceiving social interactions negatively, while being depressed is not necessarily accompanied by such negative expectations.

To understand the mechanisms of loneliness and depression, we utilized individuals’ Facebook language in addition to standardized psychometric assessments in the present study. Language on social media platforms contains rich ecological signals reflecting one’s emotions, thoughts, behavioral patterns, and traits^28^, and at the same time, can be quantified and interpreted for subsequent predictions^29^. Social media language has been shown to provide nuanced insights into and reliably predict individuals’ psychology and cognition^30,31^ as well as revealing the online sequelae of mental health conditions^32–35^. Facebook, as the most widely used social media platform in the United States^36^, provides a unique opportunity to document one’s daily life and has been used to extract linguistic features to successfully predict depression^27^, personality^31^, substance use disorder treatment outcomes^37^, and suicide risk^38^.

In the present study, we surveyed a large sample of individuals across the United States using established psychological assessments (UCLA Loneliness 3-item subscale, ULS-3)^39^ and clinical standards (Patient Health Questionnaire, PHQ-9)^40^; and found language markers significantly associated with both constructs using open-vocabulary words, phrases, and themes along with the closed-vocabulary psycholinguistic dictionary, Linguistic Inquiry Word Count 2015 (LIWC 2015)^41^. We obtain significant language markers of loneliness after controlling for age and gender, before and after controlling for depression. Our contributions are two-fold. First, we show that loneliness and depression have large overlaps in language markers of protective and risky factors, and we further elucidate unique features of loneliness through language use patterns; second, we demonstrate the predictive utility of language in assessing loneliness and depression. Our findings provide initial evidence from individuals’ Facebook language on the manifestation of loneliness and its relationship to depression, which can potentially inform future personalized interventions to reduce loneliness.

## Methods

### Participants

The current study is the secondary data analysis of the data collected from a larger study, recruited via the Qualtrics Panel. In the original study, United States-based participants received an incentive for answering a series of surveys and were invited to share access to their Facebook status updates. We obtained informed consent to access their Facebook posts and extracted all posts via the Facebook Graph API. Out of 3215 participants recruited, 3043 participants passed the attention check question. Two thousand nine hundred eighty-six participants (*M*(*SD*)_age_ = 43.24(12) year-old, 69.7% female, 63.8% have Bachelor’s or higher degrees, see Supplementary Table 1; see segmented demographics and examples of rephrased Facebook posts by depression and loneliness scores in Supplementary Table 2) who completed ULS-3, PHQ-9, and the key demographic questions (e.g., age, gender) to present study and had active accounts with more than 500 words on Facebook, were included in the data analysis. The gender in our analysis was categorized into two categories (“is female” vs. other). The original study received approval from the Institutional Review Board (IRB) of Purdue University, and the secondary data analysis in the present study was exempted by the IRB of the University of Pennsylvania.

### Materials

#### 3-item UCLA loneliness (ULS-3^39^)

The 3-item UCLA loneliness scale consists of three questions from the 20-item Revised UCLA Loneliness scale^42^ (“How often do you feel that you lack companionship?,” “How often do you feel left out?,” “How often do you feel isolated from others?”) using the 4-point Likert scoring system to capture more variance of loneliness^43,44^ (1 = “Never”, 2 = “Rarely”, 3 = “Sometimes”, 4 = “Often”). The total score of ULS-3 ranges from 3–12, with higher scores representing greater loneliness. This scale has been widely used in previous literature and displayed good reliability^45^. In the current sample, ULS-3 showed good reliability (Cronbach’s *α* = 0.81).

#### Patient Health Questionnaire-9 (PHQ-9^40^)

The PHQ-9 is a 9-item questionnaire based on the nine criteria for depression disorders in the DSM-IV (i.e., “little interest or pleasure in doing things;”). It provides the diagnosis of depression and assesses the severity of the symptoms in the past two weeks using a 4-point scale (from “Not at all” to “Nearly every day”). Previous studies have identified its great internal reliability (*α* > 0.85) and test-retest reliability^46,47^. In the current sample, PHQ-9 showed excellent reliability (Cronbach’s *α* = 0.90).

### Linguistic attributes

We characterized participants’ Facebook posts (3,459,854 Facebook posts) using two sets of language features: (a) dictionary-based psycholinguistic features, and (b) open-vocabulary topics. Following prior studies^27^, we included participants’ entire Facebook language to yield interpretable and fine-grained language variables and results.

#### Closed vocabulary

We first utilized an established language dictionary based on categories of words developed by psychologists, Linguistic Inquiry and Word Count (LIWC 2015)^41^. This top-down (theory-driven) approach has been widely used in past language research and proved to be accurate in detecting linguistic patterns associated with psychological traits such as personality^29^. We calculated the relative frequency of each LIWC category by summing up the within-participant word frequencies within each LIWC category.

#### Open vocabulary

We then used an open-vocabulary approach to extract words and phrases (1–3-grams) and used Latent Dirichlet Allocation topic modeling (LDA)^48^ to generate data-driven linguistic features called topics. We split (‘tokenized’) the Facebook posts into words, punctuation, and emoticons. Facebook posts were tokenized using *happierfuntokenizing* (DLATK/*happierfuntokenizing*, 2017), which improves over most tokenizers for tokenizing emoticons. All words used by less than 1% of users were removed from the analysis to remove uncommonly used words (outliers). This resulted in 4143 unique 1–3-grams.

The LDA generative model assumes that posts are generated by a combination of topics and that topics are a distribution of words. Since the words in a post are known, topics, which are latent variables, can be estimated through Gibbs sampling. An example of such a model is the following set of words (e.g., ‘tuesday’, ‘monday’, and ‘wednesday’), which cluster together days of the week by discovering their similar distributional properties across posts. We used an open-source set of 2000 topics trained on a corpus of over 20 million Facebook statuses^31^. We calculated the topic distribution of each user aggregated across all posts.

#### BERT embeddings

We also used Bidirectional Encoder Representations from Transformers (BERT), a pre-trained contextual word embedding model, to generate numeric vector representations of users' language. Because the BERT embedding of each word differs depending on what other words are used near it, we expect these models to capture contextual semantic information in our Facebook language data that is lost with other methods. To calculate our user vectors, we represent each word by its 10th layer in the BERT model; these vectors are averaged to produce a message embedding, and messages are finally averaged to produce user-level vector representations.

### Identifying differentially expressed language features for loneliness

We designed this as a person-level analysis and used each language feature dimension (LIWC, words/phrases, and LDA topics) as an independent variable in an OLS linear regression model to predict individuals’ loneliness with age and gender as controls, and then with depression as an additional control. By conventional linguistic analysis, we used a *p*-value of < 0.01 for LIWC and LDA topics and < 0.05 for words and phrases. All *p*-values were corrected using the Benjamini–Hochberg False Discovery Rate correction (BH-FDR correction).

### Building prediction models for loneliness using language

We evaluated how well Facebook language could predict loneliness and depression. We treated each linguistic feature set (LIWC 2015 categories, 1–3-grams, LDA topics, and BERT embeddings) as independent variables, and treated self-report loneliness and depression scores as continuous dependent variables in predictive models. Each feature set was considered independently to enable a comparative analysis of their effectiveness. The predictive model was trained using linear regression with ridge regularization, on the training set and evaluated on a test set to avoid overfitting, via cross-validation. Hyperparameter selection was performed within the cross-validation. We report Pearson’s *r* on an out-of-sample 10-fold cross-validation setting to showcase the predictive power.

### Reporting summary

Further information on research design is available in the Nature Research Reporting Summary linked to this article.

## Results

### Interoutcome survey correlations

We conducted Pearson’s correlations among age, loneliness, and depression. We found that consistent with prior work, loneliness is significantly increased with higher levels of depression but decreased with people’s age^5^. To examine the shared and unique associations between them, we conducted a series of cross-control partial correlations^49^ (Table 1). When controlling for loneliness, the correlations between age and depression remained statistically unchanged (*p* < 0.001). When controlling for depression, the correlation between loneliness and age became insignificant (*p* > 0.1). Results of Welch *t*-tests comparing the gender differences for loneliness and depression showed no effects of gender on both (both |*t* | < 1.7, *p* > 0.1).

**Table 1:** Pearson’s and partial correlations between loneliness, depression, and age.

Controlled variable	Loneliness	Depression	Age
	*r* [95% CI]	*r* [95% CI]	*r* [95% CI]
None			
Loneliness	1		
Depression	0.554 [0.53, 0.58]***	1	
Age	−0.139 [−0.17, −0.10]***	−0.256 [−0.29, −0.22]***	1
Age			
Loneliness	1		
Depression	0.541 [0.52, 0.57]***	1	
Depression			
Loneliness	1		
Age	0.004 [−0.03, 0.04]		1
Loneliness			
Depression		1	
Age		−0.218 [−0.25, −0.18]***	1

Numbers in the tables are correlation coefficients with the 95% confidence interval in square brackets: [lower bound, upper bound]. Loneliness was assessed using 3-item UCLA Loneliness Scale; Depression was assessed using Patient Health Questionnaire-9. All correlations were bivariate correlations. All *p*-values were corrected using the Benjamini–Hochberg correction.

*95% CI* 95% confidence interval, *L* lower, *U* upper.

***p < 0.001, **p < 0.01, *p < 0.05.

### Language correlates with loneliness and depression

We correlated loneliness and depression with linguistic features extracted from participants’ Facebook posts via two approaches: (a) dictionary-based closed-vocabulary (LIWC 2015)^41^, and (b) open-vocabulary-based words and phrases (1–3-grams) and Latent Dirichlet Allocation (LDA) Facebook topics^48^. We controlled for age and gender in all analyses and then added depression as an additional control.

### Loneliness and depression have large overlaps in language markers

Across all approaches, we found that loneliness has a large amount of shared risk and protective language markers with depression. That is, for the linguistic markers that were significantly correlated with loneliness, 87.1% LIWC categories (*p* < 0.01), 51.8% words and phrases (*p* < 0.05), 74.9% LDA topics (*p* < 0.01), with Benjamini–Hochberg False Discovery Rate (BH-FDR) correction, were also significantly correlated with depression. For example, among the 31 LIWC categories that were significantly correlated with loneliness (*r*_positive_ = [0.054, 0.122], *r*_negative_ = [−0.055, −0.122]), 27 were also significantly correlated with depression at the same significance level (*r*_positive_ = [0.054, 0.164], *r*_negative_ = [−0.073, −0.094], *p* < 0.01 with BH-FDR correction, see the full list of LIWC categories in Supplementary Table 3).

**Fig. 1:**
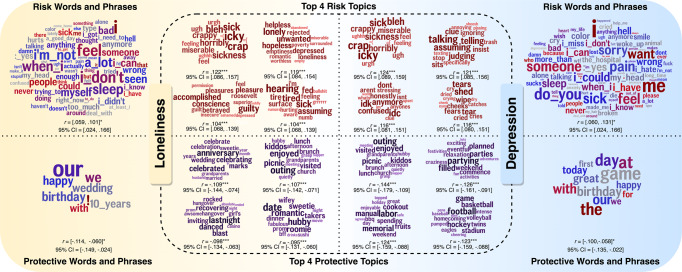
Loneliness and depression have large overlaps in language markers, illustrated by words and phrases and top four Facebook topics. All correlations in the table are controlled for age and gender. The collections of words and phrases results are present with a minimum significant level at *p* < .05. Risk = correlations with *r* > 0, protective = correlations with *r* < 0. *r* = Pearson’s correlation coefficient, the range of *r* values: [min *r*-value, max *r*-value]. 95% CI 95% confidence interval: [lower bound, upper bound]. ***p < .001, **p < .01. *p < .05. All *p*-values were corrected using the Benjamini–Hochberg False Discovery Rate correction. The font size of the word in each word cloud represents the correlation strength, the larger the size the stronger the correlation was.

Similar results were observed in open-vocabulary analyses (Fig. 1). Specifically, higher levels of loneliness and depression were both correlated with more language about negative emotions (LIWC: anxiety, anger, sad; words and phrases: ‘feel’, ‘bad’; topics about negative feelings), sickness and pain (LIWC: health; words and phrases: ‘pain’, ‘sick’; topics about pain and sickness), cognitive process (LIWC: differentiation, tentative, insight, cause; words and phrases: ‘thought’, ‘know’; topics about cognition, observation, judgment), use of first-person singular pronouns (LIWC: I; words and phrases: ‘i_don’t’, ‘i_could’; topics about low self-worth), and other themes such as sleep, present temporal orientation, risk, death, interrogatives, and fillers.

Lower levels of loneliness and depression were associated with more linguistic markers of social relationships and activities (LIWC: we, affiliation; words and phrases: ‘our’, ‘we’, ‘birthday’, ‘wedding’, ‘game’; topics about social relationships, social gatherings and activities). Words and phrases and the top four topics that were significantly associated with depression and loneliness are shown in Fig. 1, and the top 15 topics correlated with loneliness and depression with and without controlling for another are shown in Supplementary Table 4 and Supplementary Table 5, respectively.

### Linguistic markers unique to loneliness

**Fig. 2:**
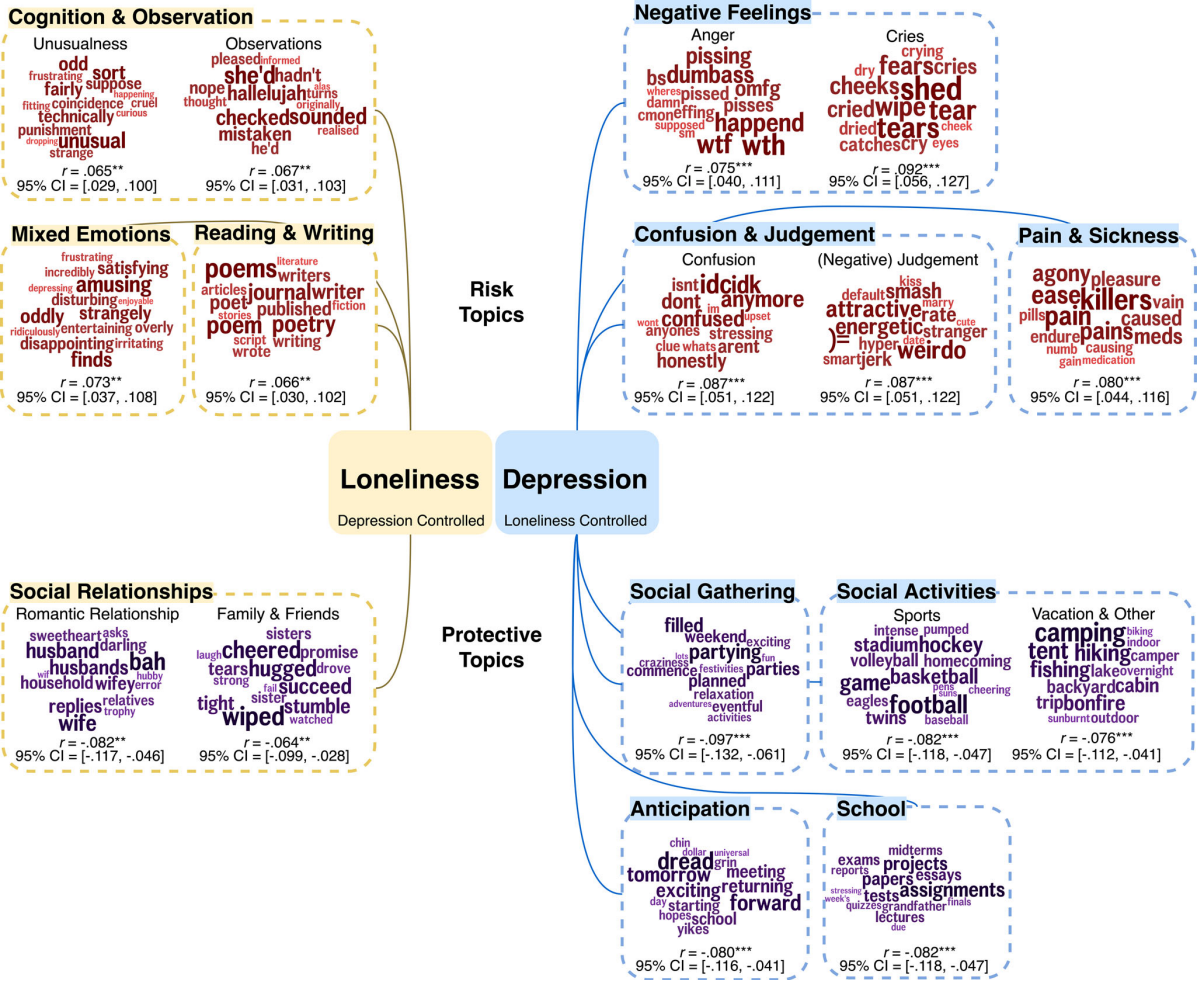
Unique Facebook topics predicting loneliness versus depression. Distinct Latent Dirichlet Allocation Topics were extracted and correlated with depression and loneliness scores, controlling for loneliness and depression scores in addition to age and gender, respectively. The top 15 correlated topics were categorized and labeled by two psychologists and presented with one example under each label to showcase the topic contents. The font size of the word in each word cloud represents the correlation strength, the larger the size the stronger the correlation was. Risk = correlations with *r* > 0, protective = correlations with *r* < 0. *r* = Pearson’s correlation coefficient. 95% CI 95% confidence interval: [lower bound, upper bound]. All correlations in the table are significant at the *p* < .01 level. ***p < .001, **p < .01. All *p-*values were corrected using the Benjamini–Hochberg False Discovery Rate correction.

After controlling for depression in addition to age and gender, there were two significant changes. First, controlling for depression made several linguistic markers of loneliness nonsignificant. That is, linguistic markers indicative of negative emotions, sickness and pain, first-person singular pronouns, and sleep were no longer correlated with loneliness. Second, after controlling for depression, correlations between loneliness and linguistic features reflecting social relationships (e.g., LIWC: we; topics about romantic relationships) remain statistically significant. Table 2 shows associations of top LIWC categories with loneliness before and after adding depression as an additional control. The top 15 correlated topics that were unique to loneliness and depression have been categorized and labeled by two psychologists and are presented with one example under each label, respectively, to showcase the topic contents in Fig. 2 (see the full list of top topics correlated with depression and loneliness with and without controlling for another in Supplementary Table 4 and Supplementary Table 5).

**Table 2:** LIWC categories associated with loneliness with and without controlling for depression.

LIWC categories			Controlled variables					
			Age, Gender			Age, Gender, Depression		
Super category	Subcategory	Example words	*r*	95% CI		*r*	95% CI	
				L	U		L	U
*Risk factors*								
Affective processes	Anxiety	fear, worry, afraid	0.096***	0.060	0.131			
	Sadness	miss, lost, sad, sorry	0.090***	0.054	0.125			
	Anger	hate, hell, stupid	0.087***	0.051	0.122			
Cognitive processes	Differentiation	but, not, if, or,	0.107***	0.071	0.142	0.061**	0.025	0.097
	Tentative	if, or, some, any, hope, may	0.104***	0.068	0.139	0.055**	0.019	0.090
	Insight	know, think, feel, find, believe	0.095***	0.059	0.130	0.061**	0.026	0.097
Total function words	Common adverbs	so, just, when	0.103***	0.068	0.138	0.057**	0.022	0.093
	Negations	not, no, don’t, can’t	0.103***	0.067	0.138			
	Auxiliary verbs	is, have, be, are, was	0.093***	0.057	0.128	0.055**	0.020	0.091
Total pronouns	1st person singular	i, my, me, I’m, I’ve	0.101***	0.065	0.136			
	Impersonal pronouns	this, it, that	0.082***	0.046	0.118			
Other grammar	Common verbs	is, have, be, are, was, will, get, do	0.086***	0.051	0.122			
Time orientations	Present focus	is, have, be, are, get	0.075***	0.039	0.110			
Personal concerns	Death	dead, died, die, war	0.075***	0.039	0.110			
*Protective factors*								
Total pronouns	1st person plural	we, our, us, let’s	−0.122***	−0.157	−0.087	−0.070***	−0.106	−0.035
Drives	Affiliation	we, love, our, friends	−0.113***	−0.148	−0.078	−0.079***	−0.114	−0.043
Social processes	Family	family, baby, mom	−0.078***	−0.113	−0.042	−0.058**	−0.094	−0.022
	Friends	friends, friend, date				−0.053**	−0.088	−0.017
Affective processes	Positive emotion	love, good, happy	−0.060**	−0.095	−0.024			
Biological processes	Ingestion	sweat, eat, water	−0.055**	−0.091	−0.019			

Top LIWC significant risk and all protective categories are present in the table. All correlations in the table are significant at *p* < 0.01 level, insignificant correlations are left blank in the table. Risk factors are those with  *r* > 0; protective factors are those with   *r* < 0. *r* = Pearson’s correlation coefficient. All *p*-values were corrected using the Benjamini–Hochberg False Discovery Rate correction.

*LIWC* Linguistic Inquiry and Word Count, English 2015 category, *95% CI* 95% confidence interval, *L* lower, *U* upper.

***p < 0 .001, **p < 0 .01.

Linguistic markers associated with a higher level of loneliness in cognition, thinking, reasoning, and observations remained after adding depression as an additional control, including LIWC categories of cognitive process (differentiations, tentative language, insight), LIWC function words (common adverbs, auxiliary verbs), the 1-gram word ‘of’ (*r* = 0.070, 95% confidence interval: [0.034, 0.106], *p* = 0.024), as well as topics about unusualness (e.g., ‘odd’, ‘unusual’, ‘strange’) and observations (e.g., ‘checked’, ‘sounded’, ‘mistaken’), writing and reading (e.g., ‘poems’, ‘journal’, ‘writer’), and mixed emotions with cognitive components (e.g., ‘amusing’, ‘oddly’, ‘disappointing’). See detailed statistics in Table 2 and Fig. 2.

Protective linguistic markers against loneliness described social relationships, including LIWC categories of 1st person plural, affiliation, family, and friends, the 1-gram word ‘our’ (*r* = −0.069, 95% confidence interval: [−0.104, −0.033], *p* = 0.024), and topics about romantic relationships (e.g., ‘husband’, ‘wife’, ‘wifey’), and (interactions with) family members and friends (e.g., ‘hugged’, ‘sister’, ‘wiped’). These linguistic features are different from those associated with depression after controlling for loneliness (Fig. 2). As it is beyond the scope of this study to describe unique factors of depression after controlling for loneliness, we present the unique topics associated with depression in Fig. 2 without further discussion.

### Facebook language predicts loneliness and depression

The predictive power of Facebook language in predicting loneliness is shown in Table 3. We found that language features outperformed age and gender in predicting loneliness and depression, but the predictive performance of both demographics and Facebook posts is lower for loneliness (*r* = 0.133, using Age and Gender; *r* = 0.201, using BERT) as compared to depression (*r* = 0.253 using Age and Gender; *r* = 0.312, using BERT).

**Table 3:** Predictive power of linguistic models in the current study.

Features	Loneliness		Depression	
	*r*	*MAE*	*r*	*MAE*
Demographics				
Age + gender	0.133	1.90	0.253	4.63
Language				
LIWC	0.190	1.87	0.283	4.58
N-grams	0.155	1.89	0.209	4.71
Topics	0.169	1.88	0.274	4.56
BERT	0.201	1.87	0.312	4.54
Demographics + Language				
Age + gender + BERT	0.204	1.87	0.321	4.52
Age + gender + LIWC + N-gram + topics + BERT	0.204	1.87	0.312	4.55

*r* = Pearson’s correlation coefficient. N-grams = words and phrases (1–3-grams). Topics = clusters of co-occurring words extracted using the Latent Dirichlet Allocation method.

*MAE* Mean Absolute Error, *LIWC* Linguistic Inquiry and Word Count, English 2015 category. *BERT* Bidirectional Encoder Representations from Transformers.

## Discussion

Our study has three main findings. Firstly, language markers associated with loneliness and depression overlap substantially (over 50% of significant linguistic markers of loneliness were also correlated with depression). Secondly, the risk for loneliness has unique linguistic markers that reflect cognitive processing of environmental information, self-oriented cognitive activities (e.g., reading and writing), and mixed emotions with cognitive components. As may be expected, linguistic references to social relationships show protective associations with loneliness. Thirdly, the variance accounted for by predictive models based on demographics and the language in Facebook posts is lower for loneliness than depression.

Loneliness shares many features with depression, as measured by surveys and language use. Our observed moderate positive correlation between loneliness and depression (*r* = 0.554) replicates other studies^5,11^. The linguistic markers positively correlated with both include negative emotions, sickness, and pain, as well as first-person singular pronouns. Linguistic markers negatively correlated with both include friends, family, and social gatherings. These correlational patterns are in line with the literature on the risky and protective linguistic markers of depression^27,33,34,50^ and loneliness^32,51,52^, and the psychological constructs (e.g., worry) associated with both^19^. This also supports the plausible effectiveness of past interventions to reduce loneliness by asking patients to focus on reducing their internal negative thoughts^20^.

The significant correlation between loneliness and age became insignificant when controlling for depression, but the correlation between depression and age remained effectively unchanged when controlling for loneliness. This suggests that depression may be the third variable driving the relationship between age and loneliness. Given the high prevalence of loneliness among the elderly with depression^4,8^, this confound should be taken into account in future work.

We observed similar patterns in language: controlling for depression rendered insignificant the majority of linguistic features correlated with loneliness, but controlling for loneliness did not impact the linguistic features correlated with depression. Because we found large overlaps between the language of loneliness and depression, to understand the shared and unique linguistic features, we looked at partial language correlations of one construct controlling for the other. While this approach foregrounds the unique language patterns associated with either construct, as has been observed by other personality researchers, we also note that the interpretation of a psychological construct may become difficult once an overlapping, closely related psychological construct is partialled out–as is the case here^53^. Effect sizes are also reduced^54^.

It is worth noting that linguistic markers of loneliness reported in past research^32,51,52^ were found to be also shared by depression in the present study, which further indicates that the impact of depression should not be overlooked in investigating loneliness. But our findings do not suggest that we should treat loneliness merely as a facet of depression just because depression shares many common features and heavily impacts loneliness. Instead, and more importantly, we found loneliness has unique manifestations in language use, which could be targets of personalized treatments.

We found that greater loneliness (unlike depression) is related to more references to greater cognitive reasoning and information processing of surroundings; while less loneliness is related to references to social relationships, after controlling for depression. Loneliness was linked to cognitive and information processing in prior cross-sectional and longitudinal surveys and neuroimaging studies^24^, serving as a protective mechanism against unpleasant social interaction and threats^25^. Lonely individuals are more likely to view the social world as threatening and pay more attention to and generate interpretations of the social environment, which may contribute to more biased expectations and attributions, and greater cognitive load in the processing and observation of social surroundings^55,56^. This potential increase in cognitive load may be supported by the correlation between LIWC cognitive process (e.g., insight and tentative categories) and loneliness. Previous findings in psycholinguistics suggest that the use of insight words reflects active processes of cognitive appraisal, and the use of tentative language reflects uncertainty^57^. A more recent study testing the relationship between cognitive processes and the use of cognitive linguistic markers from LIWC also suggests that higher cognitive load is associated with greater use of cognitive linguistic markers^58^.

We also observed that loneliness is negatively correlated with language about close relationships (i.e., romantic relationships and friends and family members) and positive social interactions related to these relationships (e.g., hugging). Although positive celebrations and gatherings could reduce both depression and loneliness, loneliness is uniquely negatively associated with social relationships rather than activities and celebrations, indicating that loneliness might be particularly driven by the lack of personal and family experiences. The above findings also mean that loneliness could be reduced through more positive social experiences and the treatment of maladaptive social cognitions^56^. Past findings on treatment have shown that the positive social interactions and relationships an individual experiences can shape their social expectations and motivations, which has been linked to lower levels of loneliness^24^; interventions designed to address maladaptive social cognition have been found to be most effective in reducing loneliness^20^.

Our findings provide consistent evidence from both closed- and open-vocabulary language analyses to support the above hypotheses, reflecting a “head versus heart” difference between loneliness and depression . That is, loneliness is positively associated with over-attention to the environment, including watchfulness towards changes, as well as mental preoccupations and reasoning, and self-directed cognitive activities like reading and writing (in the “head”). Depression, on the other hand, focuses on negative emotions, pain perception, and emotionally-focused rumination (from the “heart”).

The current study has limitations. First, the current study is correlational; the causal relationship between loneliness, depression, and linguistic features cannot be established. Second, our findings are based on a sample of adults in the United States; findings cannot easily be extended to teenagers or older adults–the two populations that most suffer from loneliness. Third, the current paper used a three-item scale for the assessment of loneliness, though with good reliability, which limits the power and accuracy in predicting and measuring loneliness. Future studies should utilize more comprehensive assessments (e.g., the standard UCLA 20-item loneliness scale^42^ and other assessments besides self-report scales, including interviews) to evaluate the levels of loneliness. Additionally, the current study only conducted a single-time assessment of depression and loneliness yet included all of the participants’ Facebook postings to extract linguistic features, which covered a wide period. The inclusion of entire timeline of Facebook posts enhances the data quantity and has been shown in the literature to produce fine-grained linguistic features, but did not foreground signal reflecting the episodic nature of depression and loneliness. Future studies should evaluate the language associated with changes in depression and loneliness over time.

When comparing the effectiveness of the current interventions to reduce loneliness, a meta-analysis^20^ found that social cognitive training interventions in social groups are more effective than other intervention types (i.e., enhancing social skills, community-based groups). Our findings also provide evidence to support this. Reflected by language use, greater loneliness is linked to preoccupation with processing environmental information and self-oriented cognitive activities. Therefore, future interventions should perhaps consider targeting clients’ perceptions, reasoning, cognitions, and relationships, especially in the context of the social environment. Potential training to reduce loneliness could focus on changing clients’ cognitive style in understanding surroundings, shifting cognitive focus from changes to the regularities in the environment, reducing self-directed activities like reading and writing, and strengthening connections to close relationships. In addition, considering the large overlap between loneliness and depression, future interventions should also consider noticing and treating clients’ other affective distress to better reduce loneliness.

### Supplementary information

Reporting Summary

Supplementary Material

## Data Availability

De-identified data necessary to reproduce the results contained in the document is available upon request from the corresponding authors. We will not, however, share individual-level Facebook data as it contains potentially identifying information about participants enrolled in the study.

## Code availability

The code used in the present paper is now available on GitHub (https://github.com/hellotingting/language-of-loneliness-wwbp).
