# Supplementary material for: Head versus heart: social media reveals differential language of loneliness from depression
Source: Npj Ment Health Res. 2022 Oct 18;1:16. doi: 10.1038/s44184-022-00014-7 (PMC10955894; doi:10.1038/s44184-022-00014-7)
Supplement: Supplementary file 2 — Supplementary Material [file 44184_2022_14_MOESM2_ESM.docx]

**Supplementary Material:**

**Head versus Heart: Social Media Reveals Differential Language of Loneliness from Depression**

**Supplementary Table 1. Demographic Information of Study Participants.** ULS-3: 3-item UCLA Loneliness Scale, range: 3-12; PHQ-9: Patient Health Questionnaire-9, range: 0-27.

| Demographics | Statistics |
| --- | --- |
| Age, Mean (SD), Median | 43.24 (12), 43 |
| Gender, Female, N (%) | 2,081 (69.7%) |
| Marital status, N (%) |  |
| Never married | 886 (29.7%) |
| Partner or married | 1592 (53.3%) |
| Separated, divorced, or widowed | 498 (16.7%) |
| ULS-3, Mean (SD) | 7.27 (2.31) |
| PHQ-9, Mean (SD) | 6.61 (5.99) |

**Supplementary Table 2. Demographics and Example Posts by Depression and Loneliness Score.** ULS-3: 3-item UCLA Loneliness Scale, range: 3-12, the cut-off for being lonely was ULS-3 total score >= 9; PHQ-9: Patient Health Questionnaire-9, score range: 0-27, the cut-off for being depressed is PHQ-9 >= 10. ^a^ Due to ethical and privacy concerns, all posts in the table were re-phrased and artificial postings.

| Demographics | Depressed & Lonely | Depressed & Not Lonely | Not Depressed & Lonely | Not Depressed & Not Lonely |
| --- | --- | --- | --- | --- |
| N (%) | 495 (16.6%) | 304 (10.2%) | 526 (17.6%) | 1,661 (55.6%) |
| Age, Mean (SD) | 39.13 (11.61) | 38.63 (11.31) | 44.07 (11.71) | 45.04 (11.83) |
| Is Female, N (%) | 343 (69.3%) | 217 (71.3%) | 373 (70.9%) | 1,149 (69.2%) |
| ULS-3, Mean (SD) | 10.04 (1.09) | 6.92 (1.22) | 9.55 (0.90) | 5.79 (1.61) |
| PHQ-9, Mean (SD) | 15.77 (4.36) | 13.70 (3.23) | 5.34 (2.68) | 2.99 (2.60) |
| Example Posts^a^ | “ugh I don't like it at all when they judge me” | “I am so sick”,  “I hate it” | “but want to connect with you” | “The greatest weekend ever!” |

**Supplementary Table 3. Associations of LIWC Categories with Loneliness and Depression.** All correlations in the table are significant at *p* < .01 level, insignificant correlations are left blank in the table. LIWC = Linguistic Inquiry and Word Count, English 2015 category. Risk = correlations with *r* > 0, protective = correlations with *r* < 0. *r* = Pearson’s correlation coefficient. All *p* values were corrected using the Benjamini-Hochberg False Discovery Rate correction.

|  | Depression | | Loneliness | |
| --- | --- | --- | --- | --- |
| LIWC Category | Pearson *r* | *p* | Pearson *r* | *p* |
| Risk Factors | | | | |
| NEGEMO | .164 | <.001 | .122 | <.001 |
| COGPROC | .114 | <.001 | .116 | <.001 |
| DIFFER | .084 | <.001 | .107 | <.001 |
| TENTAT | .090 | <.001 | .104 | <.001 |
| ADVERB | .083 | <.001 | .103 | <.001 |
| NEGATE | .113 | <.001 | .103 | <.001 |
| I | .143 | <.001 | .101 | <.001 |
| ANX | .107 | <.001 | .096 | <.001 |
| INSIGHT | .061 | .001 | .095 | <.001 |
| AUXVERB | .068 | <.001 | .093 | <.001 |
| SAD | .128 | <.001 | .090 | <.001 |
| ANGER | .126 | <.001 | .087 | <.001 |
| VERB | .089 | <.001 | .086 | <.001 |
| FUNCTION | .054 | .004 | .086 | <.001 |
| IPRON | .084 | <.001 | .082 | <.001 |
| FOCUSPRESENT | .086 | <.001 | .075 | <.001 |
| DEATH | .103 | <.001 | .075 | <.001 |
| PRONOUN | .133 | <.001 | .071 | <.001 |
| RISK | .057 | .003 | .067 | .001 |
| INTERROG | .107 | <.001 | .064 | .001 |
| COMPARE |  |  | .060 | .004 |
| BODY | .112 | <.001 | .058 | .005 |
| HEALTH | .108 | <.001 | .056 | .005 |
| CAUSE | .069 | <.001 | .056 | .006 |
| FILLER | .079 | <.001 | .054 | .010 |
| Protective Factors | | | | |
| WE | -.094 | <.001 | -.122 | <.001 |
| AFFILIATION | -.062 | .001 | -.113 | <.001 |
| FAMILY |  |  | -.078 | <.001 |
| DRIVES | -.073 | <.001 | -.069 | .001 |
| POSEMO |  |  | -.060 | .004 |
| INGEST |  |  | -.055 | .007 |

**Supplementary Table 4. Top 15 Risk and Protective Facebook Topics Associated Depression Before and After Controlling for Loneliness.** All correlations in the table are significant at *p* < .01 level. Topics: clusters of co-occurring words extracted using Latent Dirichlet Allocation method. Risk = correlations with *r* > 0, protective = correlations with *r* < 0. *r* = Pearson’s correlation coefficient. ****p* < .001, ***p* < .01. All *p* values were corrected using the Benjamini-Hochberg False Discovery Rate correction. Sorted by correlation coefficient values when controlling for age, gender, and loneliness.

| Facebook Topics | Controlled Variables | | | | | | | | | |
| --- | --- | --- | --- | --- | --- | --- | --- | --- | --- | --- |
|  | Age, Gender | | | | Age, Gender, Loneliness | | | | |  |
|  | *r* | 95% CI | | *r* | | 95% CI | | |  |  |
|  |  | L | U |  |  | L | U |  |  |  |
| Risk Factors | | | | | | | |  |  |  |
| kiss, touch, lips, eyes, hold, hand, kissed, mine, taste, soft, cheek, warm, breath, kissing, skin | .116*** | .081 | .151 | .101*** | | .065 | .136 |  |  |  |
| tears, eyes, cry, tear, smile, pain, shed, crying, cried, wipe, dry, fears, hide, smiles, brought | .116*** | .080 | .151 | .092*** | | .056 | .127 |  |  |  |
| cry, make, laugh, smile, makes, tears, happened, laughs, shoulder, crying, laughter, cope | .113*** | .078 | .148 | .089*** | | .053 | .124 |  |  |  |
| im, dont, anymore, idk, confused, whats, wont, isnt, alot, honestly, idc, mad, ive, upset, arent | .116*** | .080 | .151 | .087*** | | .051 | .122 |  |  |  |
| )=, cute, i'd, date, kiss, rate, funny, smart, random, tough, marry, attractive, hug, ugly, chill | .087*** | .051 | .122 | .087*** | | .051 | .122 |  |  |  |
| smile, make, makes, laugh, takes, cry, giggle, everytime, frown, muscles, style, extend, arm, annoys, smiling | .101*** | .065 | .136 | .085*** | | .049 | .120 |  |  |  |
| talking, people, stop, telling, talk, idea, annoying, they're, clue, count, assuming, judging, trash, honestly, texting | .121*** | .085 | .156 | .083*** | | .048 | .119 |  |  |  |
| fucking, fuck, shit, pissed, bullshit, goddamn, piss, bitch, wtf, asshole, fucked, >:, pissing, shitty, pisses | .107*** | .071 | .142 | .082*** | | .046 | .117 |  |  |  |
| fucking, fuck, shit, hate, stupid, retarded, bitch, pissed, omfg, kidding, shitty, bitches, annoying, bullshit, idiot | .108*** | .072 | .143 | .081*** | | .046 | .117 |  |  |  |
| lol, nah, haha, hahaha, kidding, hahah, hah, lmao, yah, eh, hahahaha, hmm, jk, hmmm, naw | .099*** | .063 | .134 | .081*** | | .046 | .117 |  |  |  |
| pain, hurt, pleasure, meds, killers, ease, gain, caused, hurts, pains, worse, chest, pills, numb, hurting | .114*** | .078 | .149 | .080*** | | .044 | .116 |  |  |  |
| miss, put, wanna, single, cute, ;), relationship, inbox, :p, falling, talk, kiss, yo, hug, (: | .073** | .037 | .108 | .078*** | | .043 | .114 |  |  |  |
| kill, kills, killed, murder, killing, die, swear, dead, alive, boredom, thrill, murdered, torture, destroy, shoot | .108*** | .072 | .143 | .077*** | | .041 | .113 |  |  |  |
| close, eyes, things, heart, dream, kiss, cry, everytime, greater, drift, imagine, pretend, temporary, burning, closing | .108*** | .072 | .143 | .077*** | | .041 | .112 |  |  |  |
| Protective Factors | | | | | | | |  |  |  |
| weekend, fun, ready, filled, lots, forward, exciting, planned, parties, begin, plans, partying, busy, camping, weekends | -.126*** | -.161 | -.091 | -.097*** | | -.132 | -.061 |  |  |  |
| great, lunch, nice, dinner, family, enjoyed, church, wonderful, afternoon, sunday, kids, evening, shopping, meeting, hubby | -.144*** | -.179 | -.109 | -.089*** | | -.124 | -.053 |  |  |  |
| tomorrow, school, start, ready, classes, excited, starting, orientation, summer, wednesday, college, tuesday, nervous, kindergarten, internship | -.106*** | -.141 | -.070 | -.087*** | | -.123 | -.051 |  |  |  |
| yay, excited, finally, woohoo, cap, graduation, coming, weeks, braces, dressing, picked, excitement, ordered, exciting, rehearsal | -.110*** | -.146 | -.075 | -.087*** | | -.122 | -.051 |  |  |  |
| weekend, forward, long, week, great, relaxing, spending, plans, tgif, recover, eventful, recovering, productive, enjoying, exhausting | -.101*** | -.136 | -.065 | -.087*** | | -.122 | -.051 |  |  |  |
| park, linkin, picnic, par, water, parks, theme, central, trailer, national, skate, playground, zoo, fireworks, bench | -.098*** | -.133 | -.062 | -.083*** | | -.119 | -.047 |  |  |  |
| papers, week, exams, assignments, tests, projects, due, homework, finals, finish, semester, classes, weeks, finished, writing | -.080*** | -.116 | -.045 | -.082*** | | -.118 | -.047 |  |  |  |
| game, football, tonight, watch, play, basketball, hockey, boys, season, homecoming, volleyball, baseball, intense, pumped, twins | -.123*** | -.159 | -.088 | -.082*** | | -.118 | -.047 |  |  |  |
| chicago, headed, heading, trip, tn, tennessee, leaving, visit, ohio, orleans, town, downtown, bound, wisconsin, alabama | -.105*** | -.141 | -.070 | -.082*** | | -.118 | -.046 |  |  |  |
| school, tomorrow, closed, schools, snow, kids, cancelled, district, cancel, canceled, county, delay, tomorow, school's, due | -.089*** | -.124 | -.053 | -.081*** | | -.117 | -.046 |  |  |  |
| school, starts, tomorrow, week, summer, monday, weeks, tuesday, wednesday, thursday, semester, tomorow, registration, kindergarten, yikes | -.099*** | -.135 | -.063 | -.081*** | | -.117 | -.046 |  |  |  |
| tomorrow, school, day, forward, starting, meeting, exciting, hopes, dread, returning, yikes, grin, chin | -.095*** | -.130 | -.059 | -.080*** | | -.116 | -.044 |  |  |  |
| day, today, yesterday, hoping, productive, eventful, absent, needless | -.097*** | -.133 | -.062 | -.079*** | | -.115 | -.044 |  |  |  |
| weather, warm, nice, cold, sunny, degrees, summer, hot, enjoying, loving, cool, breeze, sun, florida, warmer | -.107*** | -.142 | -.071 | -.079*** | | -.115 | -.043 |  |  |  |

**Supplementary Table 5. Top 15 Risk and Protective Facebook Topics Associated with Loneliness Before and After Controlling for Depression.** All correlations in the table are significant at *p* < .01 level, insignificant correlations are left blank in the table. Topics: clusters of co-occurring words extracted using Latent Dirichlet Allocation method. Risk = correlations with *r* > 0, protective = correlations with *r* < 0. ****p* < .001, ***p* < .01. All *p* values were corrected using the Benjamini-Hochberg False Discovery Rate correction. Sorted by correlation coefficient values when controlling for age, gender, and depression.

| Facebook Topics | Controlled Variables | | | | | | | | |  |  |
| --- | --- | --- | --- | --- | --- | --- | --- | --- | --- | --- | --- |
|  | Age, Gender | | | Age, Gender,  Depression | | | | | | |  |
|  | *r* | 95% CI | | *r* | | 95% CI | | | | | |
|  |  | L | U |  |  | L | | U | | | |
| Risk Factors | | | | | | | | | |  |  |
| finds, annoying, extremely, interesting, thinks, slightly, amusing, depressing, incredibly, entertaining, odd, confusing, kind, frustrating, fact | .089*** | .054 | .125 | .073** | .037 | | .108 | |  |  |  |
| end, beginning, ending, story, cycle, stories, ends, knowing, middle, endings, begining, beginnings, chapter, clear, circle | .061** | .025 | .097 | .069** | .033 | | .105 | |  |  |  |
| thought, didn't, guess, found, turns, wrong, knew, wasn't, wanted, realized, couldn't, checked, heard, turned, decided | .093*** | .057 | .128 | .067** | .031 | | .103 | |  |  |  |
| supposed, part, they're, aren't, we're, suppose, apparently, permanent, temporary, choked, heck, excitement, madness, life's, discover | .084*** | .048 | .120 | .067** | .031 | | .102 | |  |  |  |
| writing, write, read, story, poetry, poem, wrote, book, reading, stories, poems, written, creative, journal, writer |  |  |  | .065** | .030 | | .102 | |  |  |  |
| it's, thing, sort, isn't, odd, suppose, strange, thinks, apparently, unusual, weird, telling, happening, fact, fairly | .075** | .039 | .110 | .065** | .029 | | .100 | |  |  |  |
| today, didn't, earlier, yesterday, couldn't, ended, planned, expected, usual, started, decided, wasn't, turned, played, showed |  |  |  | .064** | .028 | | .100 | |  |  |  |
| it's, i'm, can't, there's, i've, feels, wrong, explain, breathe, fight, steel, somebody's, knife, suffer, drown | .083*** | .048 | .119 | .063** | .027 | | .099 | |  |  |  |
| change, world, changing, plans, constant, seasons, ways, attitude, somethings, growth, subject, nothing's, pace, direction, constantly | .077*** | .041 | .112 | .063** | .027 | | .099 | |  |  |  |
| feel, sick, crap, feeling, ugh, hate, feels, sucks, crappy, bleh, worse, miserable, sickness, icky, =( | .122*** | .086 | .157 |  |  | |  | |  |  |  |
| lonely, feel, feeling, sad, depressed, empty, hopeless, loneliness, romantic, helpless, unwanted, miserable, rejected, surrounded, terrible | .119*** | .084 | .154 |  |  | |  | |  |  |  |
| i'm, sick, tired, feeling, hearing, tire, fed, bullshit, assuming, hurting, numb, surface, expecting, aware, complaining | .104*** | .068 | .139 |  |  | |  | |  |  |  |
| feel, make, guilty, accomplished, pleasure, depressed, conscience, guilt, betrayed, ashamed, pleasures, permission, superior, insecure, confident | .104*** | .068 | .139 |  |  | |  | |  |  |  |
| sleep, can't, lack, deprived, nights, unable, insomnia, caffeine, catching, repeat, function, grumpy, snoring, overrated | .101*** | .065 | .136 |  |  | |  | |  |  |  |
| sleep, can't, tired, ugh, insomnia, sucks, grr, grrr, reverse, grrrr, night's, overrated, snoring, nyquil, restless | .098*** | .063 | .134 |  |  | |  | |  |  |  |
| Protective Factors | | | | | | | | | |  |  |
| wife, husband, kids, bah, wonderful, future, daughter, hubby, honey, darling, girlfriend, asks, wifey, replies, sweetheart | -.084*** | -.120 | -.048 | -.082*** | | -.117 | | -.046 | | | |
| married, man, marry, wife, single, engaged, dating, woman, men, husband, women, marriage, wives, happily, husbands |  |  |  | -.071** | | -.106 | | -.035 | | | |
| sister, made, laugh, strong, watched, friend, tears, forever, wiped, mom, succeed, cheered, tight, hugged, promise |  |  |  | -.064** | | -.099 | | -.028 | | | |
| happy, anniversary, year, years, wedding, month, celebrating, celebrate, parents, married, husband, hubby, yr, celebration, celebrated | -.109*** | -.144 | -.074 |  | |  | |  | | | |
| great, lunch, nice, dinner, family, enjoyed, church, wonderful, afternoon, sunday, kids, evening, shopping, meeting, hubby | -.107*** | -.142 | -.071 |  | |  | |  | | | |
| night, fun, blast, great, party, awesome, danced, awsome, lastnight, hangover, ended, dancing, recovering, prom, enjoyed | -.098*** | -.134 | -.063 |  | |  | |  | | | |
| night, date, dinner, great, movie, hubby, wonderful, lunch, girls, prom, movies, romantic, enjoyed, fantastic, fabulous | -.095*** | -.131 | -.060 |  | |  | |  | | | |
| birthday, happy, wishes, b-day, celebrate, celebrating, birthdays, present, celebration, cake, celebrated, mom's, belated, dad's, brother's | -.095*** | -.130 | -.059 |  | |  | |  | | | |
| day, weekend, great, labor, memorial, spending, bbq, manual, cookout, fruits | -.092*** | -.128 | -.057 |  | |  | |  | | | |
| party, birthday, bday, b-day, surprise, celebrate, st, saturday, celebrating, bash, cake, blast, celebration, graduation, planning | -.092*** | -.128 | -.057 |  | |  | |  | | | |
| happy, birthday, wishing, sister, years, wonderful, st, daughter, nephew, brother, son, turns, niece, special, celebrate | -.092*** | -.127 | -.056 |  | |  | |  | | | |
| happy, birthday, birthdays, camper, wishing, wished, happiest, endings | -.088*** | -.124 | -.053 |  | |  | |  | | | |
| wedding, married, royal, congrats, planning, reception, dress, congratulations, ring, bride, engagement, ceremony, cousin, engaged, maid | -.087*** | -.122 | -.051 |  | |  | |  | | | |
| great, night, awesome, friends, hung, amazing, guys, blast, met, hanging, hang, chilled, besties, buddies, helped | -.086*** | -.122 | -.051 |  | |  | |  | | | |
| day, happy, valentines, independence, valentine's, pi, celebrate, celebrating, advance, veterans, republic, indians | -.086*** | -.122 | -.051 |  | |  | |  | | | |
